# Editorial: Advances in machine learning methods facilitating collaborative image-based decision making for neuroscience

**DOI:** 10.3389/fncom.2023.1267489

**Published:** 2023-09-12

**Authors:** Chengjia Wang, Heye Zhang, Georgios Papanastasiou, Guang Yang

**Affiliations:** ^1^School of Mathematical and Computer Sciences, Heriot-Watt University, Edinburgh, United Kingdom; ^2^BHF Centre for Cardiovascular Science, The University of Edinburgh, Edinburgh, United Kingdom; ^3^School of Biomedical Engineering, Sun Yat-sen University, Shenzhen, China; ^4^School of Computer Science and Electronic Engineering, University of Essex, Colchester, United Kingdom; ^5^National Heart & Lung Institute, Imperial College London, London, United Kingdom; ^6^Bioengineering Department and Imperial-X, Imperial College London, London, United Kingdom

**Keywords:** machine learning, multi-modality, federated learning, heterogeneous models, dynamic convolution, transformer, IoT

The field of neuroscience has experienced rapid advancements in recent years, fueled by the increasing availability of diverse data modalities (Biswal et al., 2010) and the development of advanced algorithms for machine learning (LeCun et al., 2015). As the global burden of brain diseases continues to rise, there is an imperative need for efficient and reliable diagnostic methods. This Research Topic aims to bring together the latest research in machine learning and neuroscience to address the challenges associated with collaborative decision-making in the diagnosis and treatment of brain diseases.

The contributions featured in this Research Topic cover a wide range of topics, from federated learning (Konečnỳ et al., 2016) and model compression (Han et al., 2015) to explainable AI (Ribeiro et al., 2016) and transfer learning (Pan and Yang, 2010). These cutting-edge research efforts seek to confront the obstacles encountered in multi-center collaboration, including issues related to performance, model transferability, data privacy, and prediction rationality in modern clinical practice.

A key theme of the articles in this Research Topic is the development of novel machine-learning techniques that enhance the efficacy and effectiveness of multimodal decision-making pertinent to brain disease diagnosis. These techniques enable researchers and clinicians to leverage the wealth of knowledge and expertise available across various centers, devices, data modalities, and analysis pipelines, ultimately leading to improved patient outcomes (Vaswani et al., 2017).

Another recurring topic in this Research Topic is the importance of transparency and explainability in a realistic clinical scenario using different medical imaging techniques. As these models become increasingly complex and more significant in clinical decision-making, researchers, clinicians, and patients must comprehend the underlying mechanisms that drive their predictions. The articles in this Research Topic cast light on the latest advances in explainable AI and provide valuable insights into developing more transparent and interpretable machine learning models for computational neuroscience (Gilpin et al., 2018).

In the first paper of the Research Topic, Huang et al. conducted a study using contrast-enhanced ultrasound (CEUS) and machine learning-assisted medical image analysis for diagnosing carotid artery disease. The research found that CEUS can be utilized to assess carotid plaque stability. A significant correlation between the CEUS enhancement degree and the expression of CD147 and MMP-9 in the plaque was observed. By leveraging machine learning algorithms, medical personnel can analyze CEUS images more accurately and efficiently, leading to potentially enhanced diagnosis and treatment of carotid artery disease. The research by Huang et al. provides a solid starting point for future investigations into machine learning-assisted medical image analysis for assessing carotid plaque stability, which could have significant implications for medical imaging.

The second work, by Ouyang et al., investigated the use of dual texture features in dynamic PET imaging reconstruction. The study sought to enhance the accuracy of PET imaging and provide more detailed information about the brain's metabolic activity. The researchers used a maximum likelihood expectation maximization (MLEM) algorithm to iterate 100 times on three composite frames to obtain three prior images. They then extracted the inverse difference moment of the GGCM and the long-run low gray-level emphasis of the GLRLM of each pixel in the three prior images, generating corresponding feature images. These feature images were input into a machine learning model that was trained to predict the final image estimate. The results demonstrated that using dual texture features improved the accuracy of PET imaging reconstruction compared to using only one texture feature or no texture features at all. This new method has potential applications in various disciplines, including diagnosis and treatment planning.

The third study, by Zhu et al., proposed a new method for emotion recognition using electroencephalogram (EEG) signals based on deep learning. The authors addressed the challenge of low resolution and limited sample size of EEG images by introducing a deep network model incorporating dynamic energy features and a Bi-LSTM network. The proposed method achieved higher accuracy than previous studies, particularly in the 3-classification problem. The authors also introduced the concept of energy sequence to effectively avoid noise superposition induced by feature extraction from single-channel sequences. This study contributes to developing more accurate and efficient emotion recognition methods using EEG signals, which have potential applications in various disciplines, such as mental health diagnosis and human-computer interaction.

Dou et al. discussed the use of machine learning algorithms to classify pediatric patients with type I bipolar disorder based on structural magnetic resonance imaging in the fourth contribution. The study used a supervised learning approach, with cortical thickness and subcortical GM volume of 86 brain regions as features for the ML classification task. The feature dimensionality was reduced by extracting meaningful features, which improved the performance of the predictive model. The study found that the machine learning algorithms achieved high accuracy in classifying pediatric patients with type I bipolar disorder, indicating their potential as a diagnostic instrument for this condition. This study emphasizes the promise of machine learning algorithms in medical imaging analysis and their potential to improve diagnosis and treatment outcomes for various conditions.

The last paper, authored by Li et al., presented a machine learning approach to improve the quality of brain MRI images acquired under clinical protocols. The proposed method employed deep attention super-resolution, a neural network that can learn to reconstruct high-resolution images from low-resolution inputs. The authors trained their model on a large dataset of brain MRI scans and evaluated its efficacy on both synthetic and real-world data. They found that their method outperformed extant state-of-the-art techniques in terms of both visual quality and quantitative metrics. The authors suggest that their approach could have significant implications for clinical neuroimaging, as it could enable more accurate diagnosis and treatment planning for patients with neurological disorders.

In conclusion, this Research Topic highlights the optimistic advancements in machine learning techniques that are poised to revolutionize how we diagnose and treat brain diseases. By fostering collaboration and addressing the challenges associated with multicenter research, these innovative approaches hold the potential to substantially improve patient outcomes and contribute to the ongoing global struggle against brain diseases. We would like to congratulate all the authors and reviewers for their invaluable contributions and insights that made this Research Topic possible.

## Author contributions

CW: Conceptualization, Data curation, Formal analysis, Investigation, Methodology, Validation, Visualization, Writing—original draft, Writing—review and editing. HZ: Conceptualization, Investigation, Resources, Supervision, Writing—review and editing. GP: Data curation, Formal analysis, Investigation, Software, Visualization, Writing—review and editing. GY: Conceptualization, Funding acquisition, Investigation, Project administration, Resources, Supervision, Writing—review and editing.
